# Impact of data resolution on three-dimensional structure inference methods

**DOI:** 10.1186/s12859-016-0894-z

**Published:** 2016-02-06

**Authors:** Jincheol Park, Shili Lin

**Affiliations:** Department of Statistics, Keimyung University, 1095 Dalgubeol-daero, Dalseo-gu, Daegu 42601, South Korea; Department of Statistics, The Ohio State University, 1958 Neil Avenue, Columbus, OH USA

**Keywords:** Data resolution, Excess of zeros, Correlated read counts

## Abstract

**Background:**

Assays that are capable of detecting genome-wide chromatin interactions have produced massive amount of data and led to great understanding of the chromosomal three-dimensional (3D) structure. As technology becomes more sophisticated, higher-and-higher resolution data are being produced, going from the initial 1 Megabases (Mb) resolution to the current 10 Kilobases (Kb) or even 1 Kb resolution. The availability of genome-wide interaction data necessitates development of analytical methods to recover the underlying 3D spatial chromatin structure, but challenges abound. Most of the methods were proposed for analyzing data at low resolution (1 Mb). Their behaviors are thus unknown for higher resolution data. For such data, one of the key features is the high proportion of “0” contact counts among all available data, in other words, the excess of zeros.

**Results:**

To address the issue of excess of zeros, in this paper, we propose a truncated Random effect EXpression (tREX) method that can handle data at various resolutions. We then assess the performance of tREX and a number of leading existing methods for recovering the underlying chromatin 3D structure. This was accomplished by creating in-silico data to mimic multiple levels of resolution and submit the methods to a “stress test”. Finally, we applied tREX and the comparison methods to a Hi-C dataset for which FISH measurements are available to evaluate estimation accuracy.

**Conclusion:**

The proposed tREX method achieves consistently good performance in all 30 simulated settings considered. It is not only robust to resolution level and underlying parameters, but also insensitive to model misspecification. This conclusion is based on observations made in terms of 3D structure estimation accuracy and preservation of topologically associated domains. Application of the methods to the human lymphoblastoid cell line data on chromosomes 14 and 22 further substantiates the superior performance of tREX: the constructed 3D structure from tREX is consistent with the FISH measurements, and the corresponding distances predicted by tREX have higher correlation with the FISH measurements than any of the comparison methods.

**Software:**

An open-source R-package is available at http://www.stat.osu.edu/~statgen/Software/tRex.

**Electronic supplementary material:**

The online version of this article (doi:10.1186/s12859-016-0894-z) contains supplementary material, which is available to authorized users.

## Background

The three-dimensional (3D) organization of a genome is closely linked to its biological functions; therefore, it is important to gain a full understanding of the genomic structure. In recent years, assays developed to identify long-range chromatin interactions genome-wide, coupled with next generation sequencing technology, revolutionize research in genomics and epigenetics. The most well-known assay for detecting chromatin interaction, Hi-C [1], produces data that identify pairs of fragments in close proximity to each other in the cell nucleus, and are commonly organized into a two-dimensional matrix (known as contact matrix) of contact counts. In addition to Hi-C, other assays for detecting genome-wide long-range interactions have also been developed, such as ChIA-PET [2], TCC [3], and single-cell Hi-C [4]. Most recently, in-situ Hi-C was debuted, achieving 1 Kb resolution with 4.9 billion contacts [5]. Furthermore, it shortens the experimental time considerably from the original Hi-C experiment.

A number of analytical approaches have been proposed to recapitulate the underlying 3D structure, with most of them developed for Hi-C data. These approaches can generally be classified into optimization based and modeling based. Most of the optimization-based approaches are two-step procedures. The idea is to first translate each pairwise contact count into a distance using a biophysical property, essentially stating an inverse relationship. One then obtains a consensus 3D structure by minimizing some objective function, such as the total “differences” between the translated distances and those inferred from the hypothesized 3D architecture [6–8]. ChromSDE [9] is an example in this category, but it also estimates the degree of the inverse relationship in addition to the 3D coordinates in the optimization step. Further, the distances between pairs with 0 contact frequencies are penalized so that they are more separated in the 3D space. ShRec3D [10] also falls into this category, except that in the first step, the contact counts are converted to distances by not just applying the inverse relationship of the biophysical model, but by also finding the “shortest path” connecting two nodes on a weighted graph. Many of the optimization methods are based on metric or non-metric multi-dimensional scaling to minimize the objective function [8, 10, 11].

Modeling-based approaches, on the other hand, are based on probability models that describe the relationship between the contact counts with the 3D physical distance. The contact counts are modeled either by a normal distribution to account for variability in the estimation [12] or by a Poisson distribution, as in BACH [13] and PASTIS [14], with its intensity parameter assumed to be related to the physical distance by an inverse relationship. Statistical inferences on the 3D structure (together with other model parameters) are made either by maximum likelihood [14] or through casting the problem into a Bayesian framework [12, 13]. Although independence assumption is not needed for optimization-based methods, this assumption is central for existing model-based methods. This leads to the concern that these models may provide a poor fit to the data as dependency among frequencies in the contact matrix are expected. Furthermore, methods that rely on the Poisson distribution fail to capture potential over-dispersion in sequencing data.

Most of the methods discussed above were proposed and tested with dilute Hi-C data at 1 Mb resolution for human lymphoblastoid cell line [1] or at 40 Kb resolution for mouse embroyonic stem cell line [15]. As discussed above, with the advancement of the technology, higher and higher resolution data are being produced at a faster rate. For a given resolution, regardless of whether it is high or low, the data are organized into a 2D matrix of contact counts. However, as the resolution gets higher and higher, the 2D matrix gets sparser and sparser, that is, with a higher proportion of zeros in the entries of the matrix. For example, for the human lymphoblastoid cell line GM12878 [5], the proportions of zeros at resolution 1 Mb, 50, 25, 20, and 10 Kb are approximately 0 *%* (<1 *%*),10,20,30, and 60 *%*, respectively, for intra-chromosomal data. As such, questions arise as to whether existing methods tested for lower resolution data are still appropriate and effective for analyzing higher resolution data, and what maybe the likely impact of higher resolution on the methods. For example, as mentioned above, ChromSDE [9] takes special care of zero contact counts, and therefore it would be of interest to evaluate whether it continues to perform well when challenged with a large proportion of zeros. On the other hand, for a model-based method such as those relying on the Poisson distribution, the underlying distribution may no longer be adequate for modeling data with excess of zeros. Specifically, if the proportion of zeros is much higher than the theoretical probability of getting a zero for a Poisson distribution that otherwise fits the non-zero frequency counts, then the model will be a poor fit for the data that include the zeros. A recently proposed truncated Poisson Architecture Model (tPAM) is an attempt to address this issue, but its appropriateness for higher resolution data with a majority of contact counts being zeros (e.g. at 10 Kb resolution) has not been evaluated. More seriously, as with the other model-based methods discussed above, tPAM also requires the independence assumption and fails to accommodate overdispersion.

In this article, we propose a truncated Random effect EXpression (tREX) model, which not only uses a truncated distribution to accommodate excess of zeros in higher resolution data, but also adds a random effect component into the model for counts. Thus tREX is expected to be robust to resolution specification, takes dependencies between contact counts into consideration, and addresses the issue of over-dispersion. By doing so, tREX can achieve greater consistency with observed data. To thoroughly investigate the performance of tREX and compare it with a number of current methods, we carried out an in-silico study. We also applied all methods to a human lymphoblastoid cell line Hi-C dataset for which FISH measurements are available to substantiate estimation accuracy.

## Results

We first describe the design and results from an in-silico study that generates data from a known 3D structure that serves as the “gold standard”. A total of 30 different (zero-inflated) Poisson contact frequency intensity models are considered. These results are based on synthetic datasets to allow for controlled settings to test methods and quantify relative merits. We emphasize the impact of resolution on the methods throughout the presentation of the results. Results for the application to a human lymphoblastoid cell line Hi-C dataset are presented following those for the in-silico study.

### An in-silico study

Using an existing estimated structure [16] as the “gold standard”, we consider several scenarios that mimic various data resolutions. This 3D structure is selected as it is estimated from real data and as it depicts two topologically associated domains (TADs) (Fig. 3(a)), an important feature for gauging the relative performance of the methods subjected to the “stress test”. Two versions of the random effect model (NRE and ST) and three sets of model parameters are used to generate the contact matrix. Moreover, five different proportions of zeros among contact counts, roughly representing five levels of data resolution, are considered: 0 % (1 Mb), 10 % (50 Kb), 20 % (25 Kb), 30 % (20 Kb), 60 % (10 Kb). This setup leads to a total of 30 simulation settings. Detailed data simulation process is described in the Methods section. In addition to tREX, the following methods are also subjected to the stress test: tPAM [16], BACH [13], PASTIS [14], ShRec3D [10], and ChromSDE [9], with the first three being model-based methods like tREX, and the remaining two being optimization based. Three aspects are evaluated: (1) estimation accuracy of the 3D coordinates of the estimated structure, (2) consideration of how well the two TADs are preserved in the estimated structure, and (3) computational time.

#### Estimation accuracy of 3D coordinates

We first discuss estimation accuracy of the 3D coordinates using two criteria: RMSD and correlation. Briefly, RMSD first computes the squared deviations between the estimated 3D coordinates and the generating coordinates, averages them, and takes the square root of the average. A more detailed description is provided in the Methods section. Correlation, on the other hand, simply computes the Pearson correlation coefficient between the estimated and the true coordinates. Both criteria measure estimation accuracy, but with emphases from two different angles; thus both are good measures of performance of a method.

**Fig. 1:**
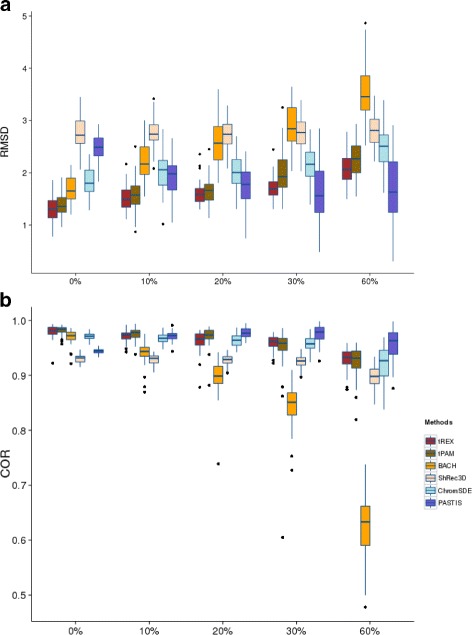
Boxplots for comparing 3D estimation accuracy of six methods under NRE model and parameter setting (*β*_0_,*β*_1_,*γ*_1_,*γ*_2_) =(3,−0.434,0.3,0.3). The comparison are for data simulated from the NRE model based on two criteria: (**a**) RMSD, and (**b**) Correlation. For each resolution/percent zeros, the six boxplots are for tREX, tPAM, BACH, ShRec3D, ChromSDE, and PASTIS, in that order

We present the results for data simulated under one set of model parameters. First we consider the NRE model. For each method and each resolution, we summarize the results across all replications in a boxplot, one for RMSD and one for correlation. The results for all methods and all resolutions (represented by different percentages of zeros in the data) are presented in Fig. 1. From these plots, one can see that BACH (third boxplot under each resolution) is affected the most by resolution, with both its RMSD increasing (Fig. 1(a)), while its correlation decreasing exponentially (Fig. 1(b)). This is not surprising as BACH was not specifically proposed for data at higher resolution and thus could not handle an excess of zeros in the contact matrix. Although the two truncated model-based methods, tREX and tPAM, appear to be slightly affected by resolution, they perform much better than BACH, at all levels of resolution, with tREX edging out tPAM consistently under the RMSD criterion. This is expected since the truncated Poisson model is specifically designed to be robust to the proportion of zeros. On the other hand, the optimization-based method, ShRec3D, does not seem to be affected by resolution, with RMSD and correlation stay fairly constant through out the entire range, but its performance is inferior to tREX and tPAM uniformly across all levels of resolution. Instead of being similar to its sibling optimization method, the behavior of ChromSDE is in fact quite similar to the modeling-based methods tREX and tPAM: as resolution increases, its performance deteriorates slightly. This feature may be due to its special handling of the zero counts as discussed above. The Poisson-based likelihood inference method, PASTIS, on the other hand, has rather unpredictable behaviors. The RMSD generally decreases but then increases very slightly as the data resolution increases, contrary to the results of all the other methods. This behavior is also observed for the correlation measure. Therefore, for higher resolution data, PASTIS may slightly outperform tREX, even though tREX is much better at lower resolution. Nevertheless, the variability of the results from PASTIS increases dramatically (i.e. having wider boxes in the boxplots) with the increase of resolution, casting a great deal of uncertainty about its results. Overall, we see that all methods maintain over 90 % correlation across all resolution, except for BACH, whose correlation drops down to about 65 % (on average) with about 10 Kb resolution.

**Fig. 2:**
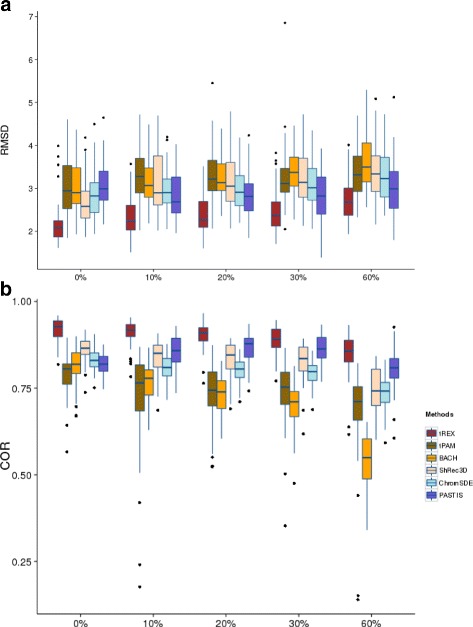
Boxplots for comparing 3D estimation accuracy of six methods under ST model and parameter setting (*β*_0_,*β*_1_,*γ*_1_,*γ*_2_)=(3,−0.434,0.3,0.3). The comparison are for data simulated from the NRE model based on two criteria: (**a**) RMSD, and (**b**) Correlation. For each resolution/percent zeros, the six boxplots are for tREX, tPAM, BACH, ShRec3D, ChromSDE, and PASTIS, in that order

The results for the ST models, given in Fig. 2, paints a clearer picture of the better performance of tREX. For the RMSD criterion (Fig. 2(a)), all methods appear to be only slightly impacted by resolution, with tREX having smaller RMSD compared to the other methods, which all seem to perform similarly (PASTIS is slightly better among them for higher resolution data, though). With respect to correlation (Fig. 2(b)), we can see that, like the data simulated under the NRE model, BACH is once again affected by resolution the most, with its correlation dropping down to only barely above 50 % with data at about 10 Kb resolution. In contrast, the correlation for tREX stays over 90 %, with ShRec3D and PASTIS (at higher resolution) being the best among the rest of the methods. These observations generally hold for the other two sets of model parameters (Additional file 1: Figures S1–4).

**Fig. 3:**
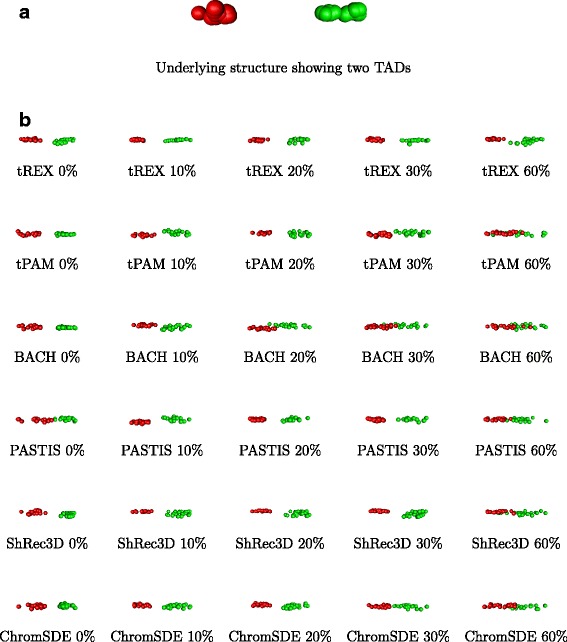
Underlying 3D structure and its estimates. **a** The 3D structure depicted was used for simulating data in the in-silico study. The red and green balls denote two topologically associated domains that are well separated in the 3D space. **b** The estimated structures depict results from one representative replicate simulated under the ST model. For each of the six methods considered, there are five estimated structures, each for the five levels of resolution/percent zeros

In summary, tREX achieves consistently good performance in all settings. Although some of the other methods may perform better at a particular resolution with a particular setting of model or parameters, tREX is always among the best (and being the best most often) in all 30 combinations of model, parameter setting, and resolution level under both evaluation criteria.

Although Figs. 1 and 2 provide clear visualizations of the results, we further quantify the performances by comparing tREX’s results with those from each of the other methods by carrying out Wilcoxon signed-rank tests for the same model and parameter settings. The results (*p*-values) are presented in Table 1. The top segment of the table (Table 1(A)) provides the *p*-values for the NRE model. These results provide formal statistical confirmation for our observations from Fig. 1: tREX has significantly smaller RMSD and significantly larger correlation than BACH and ShRec3D, but not necessarily uniformly significantly better than tPAM, PASTIS, or ChromSDE under both criteria for all resolutions. For the data simulated from the ST model, the bottom segment of the table (Table 1(B)) shows, without a doubt, that tREX has statistically significantly smaller RMSD and larger correlation than any of the other methods across all levels of resolution. The results for the other two sets of model parameters are similar (Additional file 1: Tables S1 and 2).

**Table 1:** *P*-values of Wilcoxon signed-rank tests comparing the performance of tREX with each of the comparison methods for the model with parameters (*β*_0_,*β*_1_,*γ*_1_,*γ*_2_)=(3,−0.434,0.3,0.3)

		Resolution/Percent zeros				
Criterion	Method	0 %	10 %	20 %	30 %	60 %
(A). NRE Model						
RMSD	tPAM	8.0×10^−3^	1.2×10^−2^	9.6×10^−2^	1.1×10^−7^	1.3×10^−7^
	BACH	5.6×10^−10^	4.1×10^−10^	3.8×10^−10^	3.8×10^−10^	3.8×10^−10^
	PASTIS	3.8×10^−10^	2.7×10^−7^	5.5×10^−3^	8.9×10^−1^	9.9×10^−1^
	ShRec3D	3.5×10^−15^	3.8×10^−10^	3.8×10^−10^	3.8×10^−10^	3.8×10^−10^
	ChromSDE	4.9×10^−10^	5.6×10^−10^	2.7×10^−9^	2.7×10^−9^	5.1×10^−9^
Correlation	tPAM	7.6×10^−1^	9.9×10^−1^	9.9×10^−1^	5.5×10^−2^	1.0×10^−1^
	BACH	1.5×10^−6^	1.3×10^−9^	8.0×10^−10^	3.8×10^−10^	3.8×10^−10^
	PASTIS	9.0×10^−10^	4.5×10^−1^	9.9×10^−1^	9.9×10^−1^	9.9×10^−1^
	ShRec3D	3.5×10^−15^	3.8×10^−10^	1.0×10^−9^	7.1×10^−10^	1.7×10^−9^
	ChromSDE	3.6×10^−7^	1.3×10^−3^	6.2×10^−1^	1.9×10^−1^	5.7×10^−2^
(B). ST Model						
RMSD	tPAM	4.9×10^−9^	1.9×10^−9^	1.2×10^−9^	2.2×10^−8^	3.6×10^−9^
	BACH	8.5×10^−10^	1.5×10^−8^	2.9×10^−9^	2.1×10^−9^	7.2×10^−9^
	PASTIS	6.3×10^−10^	1.0×10^−5^	3.1×10^−7^	2.3×10^−4^	6.3×10^−4^
	ShRec3D	4.3×10^−7^	1.5×10^−6^	9.3×10^−7^	8.4×10^−7^	9.6×10^−9^
	ChromSDE	1.2×10^−8^	2.0×10^−8^	1.2×10^−8^	1.2×10^−8^	4.6×10^−8^
Correlation	tPAM	3.8×10^−10^	3.8×10^−10^	4.1×10^−10^	3.8×10^−10^	1.2×10^−9^
	BACH	3.8×10^−10^	3.8×10^−10^	3.8×10^−10^	3.8×10^−10^	4.1×10^−10^
	PASTIS	3.8×10^−10^	1.5×10^−8^	4.9×10^−6^	1.5×10^−4^	1.5×10^−5^
	ShRec3D	1.0×10^−9^	3.1×10^−10^	5.3×10^−9^	3.6×10^−8^	8.6×10^−10^
	ChromSDE	4.1×10^−10^	3.8×10^−10^	4.1×10^−10^	4.3×10^−10^	3.2×10^−9^

The results for the NRE model are given in the top segment (A) and those for the ST model are given in the bottom segment (B)

#### TAD preservation

We next investigate the performance of the comparison methods for preserving the two topologically associated domains in the underlying structure. As we can see from the underlying structure depicted in Fig. 3(a), the two domains (red and green balls, respectively) are well separated, with loci within each domain cluster together tightly. As such, we use average silhouette width [17] as a measure of how well the domains are separated. Specifically, we compute the ratio of the average silhouette width of the estimated 3D structure to that of the underlying structure, and a larger value is indicative of better separation of the two TADs. As we can see from the results of one parameter setting presented in Table 2, for data simulated under the NRE model, tREX, tPAM, and ChromSDE are similar in their ability to identify the two topological domains, with their ability slightly decreased with higher resolution data. In contrast, ShRec3D performed steadily across all levels of resolution, but its ability to separate the two TADs is not as good as the other three methods just discussed. BACH, on the other hand, sees its performance degraded quickly as the resolution gets higher. As seen earlier, the performance of PASTIS is rather unpredictable. It does not performed as well as the other methods for low resolution data, but its performance gets much better with the increase of resolution, especially for mid-level resolution data. For data simulated under the ST model, tREX has higher average silhouette ratios across all levels of resolution compared to the other five methods, among them, BACH continues to perform the worst as resolution increases. Results for the other two parameter settings are similar (Additional file 1: Tables S3 and 4). As an example, we display in Fig. 3(b) the structures estimated by all six comparison methods across the resolutions for a representative dataset simulated under the ST model. As we can see from the figure, when the resolution is low (1 Mb or 0 % zeros), all methods but PASTIS recover the two TADs in the underlying structure as shown in Fig. 3(a). However, as the resolution increases, one can see that the two TADs are preserved only in the structure predicted by tREX at the highest resolution considered (10 Kb or 60 % zeros). Once again, BACH has the worst performance, which started to have trouble separating the two TAD at the 25 Kb (20 % zero contact counts) resolution.

**Table 2:** Average silhouette width ratio for the model with parameters (*β*_0_,*β*_1_,*γ*_1_,*γ*_2_)=(3,−.434,0.3,0.3). Each number represents the ratio of the average silhouette width of the estimated structure and the average silhouette width of the underlying 3D structure

		Resolution				
Model	Method	0 %	10 %	20 %	30 %	60 %
NRE	tREX	0.875	0.847	0.830	0.813	0.757
	tPAM	0.893	0.870	0.861	0.823	0.752
	BACH	0.851	0.756	0.665	0.597	0.405
	PASTIS	0.756	0.875	0.906	0.926	0.900
	ShRec3D	0.737	0.732	0.727	0.722	0.683
	ChromSDE	0.865	0.849	0.837	0.816	0.735
ST	tREX	0.757	0.746	0.734	0.716	0.670
	tPAM	0.623	0.592	0.570	0.563	0.540
	BACH	0.626	0.570	0.532	0.493	0.358
	PASTIS	0.617	0.694	0.720	0.717	0.646
	ShRec3D	0.686	0.667	0.658	0.651	0.575
	ChromSDE	0.643	0.643	0.630	0.616	0.553

#### Computational time

Computational feasibility is a major concern for genomic data, but the concern is even greater for chromatin interaction data as the size of the data is *O*(*n*^2^) when there are *n* genomic loci, an order of magnitude increase compared to analysis of linear chromosomal data. For model-based methods (except PASTIS), the computational time is typically longer as most methods are based on Markov chain Monte Carlo (MCMC) sampling, a computationally intensive technique. Optimization-based methods are usually much faster. Among modeling-based methods, those based on a truncated distribution (e.g. tREX and tPAM) will have an advantage as its computational cost can be greatly reduced for data with a higher percentage of zeros through excluding the zero counts. To illustrate this, we present some computational time analysis in Table 3 for one set of model parameters.

**Table 3:** Typical running time ^∗^ (in minutes) for completing the 3D reconstruction for a simulated dataset

	Resolution/Percent zeros				
Methods	0 %	10 %	20 %	30 %	60 %
tREX	7.6	7.3	6.5	5.3	3.3
tPAM	6.8	6.7	5.3	4.7	2.9
BACH	7.9	7.9	7.9	7.9	7.9
PASTIS	1.0	1.0	1.0	1.0	1.0
ShRec3D	1.0	1.0	1.0	1.0	1.0
ChromSDE	2.4	2.6	2.3	1.9	2.1

^*^For PASTIS and ShRec3D, the typical computational time is less than 1 min. However, PASTIS took several min to run for a few of the replications

As we can see, ShRec3D and PASTIS run much faster than the rest of the methods for each simulated data set. ChromSDE comes in next, although not an order of magnitude better as in ShRec3D and PASTIS. BACH’s running time is constant regardless of the percentage of zero contact counts. On the other hand, tREX and tPAM’s computational time reduces as the percentage of zeros increases since the number of data points needed to be analyzed decreases. ChromSDE appears to have a similar trend, perhaps due to its special way of handling zero count data. It is noted, though, that the computational time presented in Table 3 is simply an illustration on how the percentage of zeros may affect different methods differently. Indeed, an increase in resolution typically lead to an increase in the number of zeros in the contact matrix. However, the dimension of the contact matrix also increases for higher resolution data. As such, the decrease in computational time for tREX and tPAM with an increase in the percent of zeros does not mean that tREX and tPAM will take less time to analyze higher resolution data.

### Analysis of human lymphoblastoid Hi-C data

We applied tREX to the Hi-C data produced by [1]. In fact, there are two Hi-C experiments performed on the same karyotypical normal human lymphoblastoid cell line, which are combined into a single data set in our analysis given their high reproducibility [1]. For comparisons, we also run the other methods, tPAM, BACH, PASTIS, ShRec3D, and ChromSDE. We focused on chromosome 14 and 22, as experimental validation data based on Fluorescence In Situ Hybridization (FISH) are available for several pairs of loci on these two chromosomes and are publicly available [1]. Specifically, [1] discussed interesting features of spatial interactions, based on the FISH measures, among 4 loci on chromosome 14 (*L*_1_, *L*_2_, *L*_3_, and *L*_4_, located in that linear order) and 4 loci on chromosome 22 (*L*_5_, *L*_6_, *L*_7_, and *L*_8_, in that linear order) using the FISH experiment. In particular, the spatial 3D distance between *L*_2_ and *L*_4_ was observed by the FISH experiments to be smaller than that between *L*_2_ and *L*_3_, despite the fact that *L*_2_ is farther apart from *L*_4_ than from *L*_3_ in terms of their linear 1D distances. A similar observation was made for (*L*_6_,*L*_7_,*L*_8_), in that the spatial 3D distance between *L*_6_ and *L*_8_ is smaller than that between *L*_6_ and *L*_7_. The resolution used is 1 Mb, which leads to 89 loci in chromosome 14 and 36 loci in chromosome 22, that is, a total of 125 loci for a join analysis of both chromosomes.

To make it possible to compare results across different methods with the FISH measurements due to different scaling factors, we first standardize all the distances (see Methods). For greater ease of digesting the information, we calculated, for each of the 9 pairs of loci for which FISH measurements of distances are available, the absolute difference between the median of the FISH measurements and the median from each of the methods (Table 4). From the table, we can see that tREX has the smallest average absolute difference from FISH (last column of Table 4). ShRec3D’s estimated distances can sometimes be fairly different from those of FISH; for example, all four distance estimates on chromosome 22 are outside of the middle 50 % of the FISH measurements, with one being outside of the range of all 100 measurements. This is reflected in its average difference from FISH being more than 3 times of that for tREX (Table 4). The other optimization-based method, ChromSDE, performed better, but still, all four distance estimates on chromosome 22 are also outside of the middle 50 % of the FISH measurements resulting in its average difference from FISH being more than 2 times of that for tREX (Table 4). Finally, other than ShRec3D, all methods predicted the spatial distance between L2 and L4 to be shorter than that between L2 and L3, and that between L3 and L4, consistent with the FISH results. For ShRec3D, its estimated distance between L2 and L4 is indeed shorter than L2 and L3 but longer than L3 and L4. For the distance between L6 and L8 compared to L6 and L7, all methods provided consistent predictions as FISH.

**Table 4:** Absolute difference between median of FISH measurements and median of estimates for each pair of loci and each method

	Absolute median difference									
Method	L12	L13	L23	L24	L34 ^*a*^	L56	L57	L67	68	Average ^*b*^
tREX	0.083	0.113	0.019	0.176	0	0.017	0.070	0.102	0.155	0.082
tPAM	0.196	0.211	0.152	0.218	0	0.125	0.149	0.084	0.155	0.143
BACH	0.031	0.200	0.175	0.026	0	0.099	0.081	0.118	0.132	0.096
ShRec3D	0.036	0.018	0.354	0.448	0	0.607	0.421	0.456	0.426	0.307
ChromSDE	0.033	0.200	0.191	0.150	0	0.262	0.240	0.295	0.284	0.184
PASTIS	0.063	0.015	0.429	0.136	0	0.129	0.132	0.182	0.203	0.143

^*a*^By design, we standardized all distances so that the median distance between loci *L*_3_ and *L*_4_ is 1 for all the methods (including FISH). Thus, the difference between each method and FISH is zero

^*b*^Average is averaging over all pairs

**Fig. 4:**
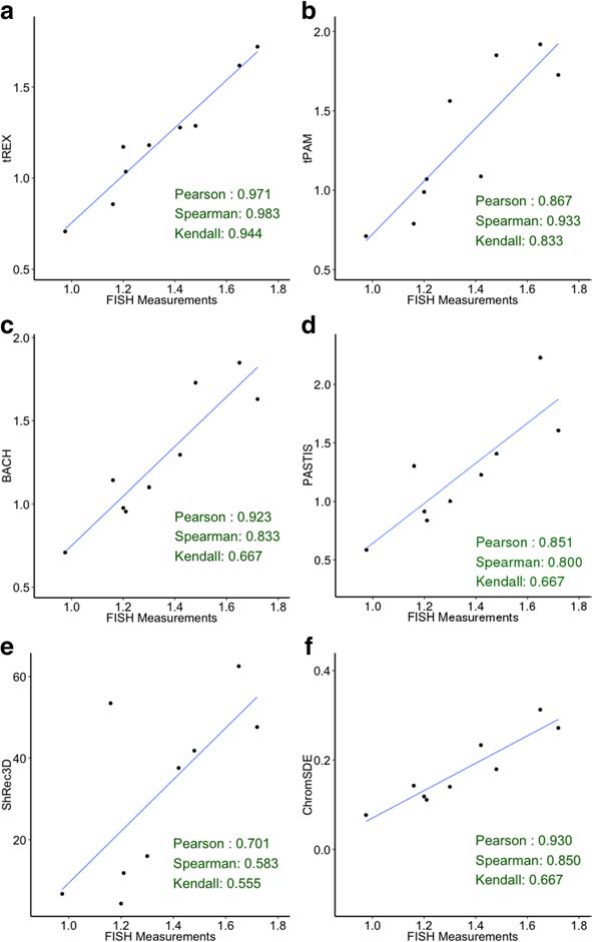
Results from analysis of human lymphoblastoid cell line Hi-C data. These plots depict correlation between the median distances from the estimates and the FISH measurements. The nine points on each plot corresponds to the nine pairs of loci. **a** tREX; (**b**) tPAM; (**c**) BACH; (**d**) PASTIS; (**e**) ShRec3D; and (**f**) ChromSDE

The observations of the relative performance of the methods are also confirmed by the correlation plots of the estimates with the FISH measurements (Fig. 4(a–f)). We considered three measures of correlation, Person’s, Spearman, and Kendall, with the latter two more suited for a non-linear relationship. All three correlations confirm that tREX performed the best overall, with the highest correlation among all methods; even the smallest correlation, Kendall’s correlation, is at 94 %. In comparison, the Kendall’s correlation can be quite low for most of the other measures: 83, 67, 67, 56, and 67 % for tPAM, BACH, PASTIS, ShRec3D, and ChromSDE, respectively. On the other hand, Pearson’s correlation is the highest for most of the method: 97, 87, 92, 85, 70, and 93 % for tREX, tPAM, BACH, PASTIS, ShRec3D, and ChromSDE, respectively.

## Discussion and conclusions

Three-dimensional organization of a genome has gained a great deal of attention in recent years. Because the structure is intimately linked to the biological functions of the genome, especially in long-range gene regulation, it is important to gain a greater understanding of the structure so that its relationship with key genomic features, such as histone marks, can be ascertained. The experimental data, organized into a 2D matrix, has special features, the most important ones include correlation among contact counts in the 2D matrix and the high proportion of zero contact counts (i.e. the matrix being sparse). To address these issues, in this paper, we propose tREX that takes correlation between read counts into consideration. In addition, over-dispersion, known to exist in next generation sequencing read count data, is also accommodated in tREX. The most important feature of tREX is that it is robust to resolution specification.

An in-silico study demonstrates these properties by showing that tREX is much more data consistent regardless of the resolution level and the underlying model from which the data were simulated. The study further shows that tREX performs at least as well as any of the current state-of-the art methods, both optimization-based and modeling-based, and in fact outperforms in most of the scenarios considered. Even when the data were simulated from the Poisson model (especially those with no zero-inflation, which matches exactly with the analysis model for tPAM, BACH, and PASTIS), tREX was still among the top performers. When correlation among contact frequencies was introduced, tREX’s advantage was clearly seen, as it was not only able to take dependencies into account, but also to accommodate overdispersion introduced by the correlation. In all, a total of 30 combinations of random effect, model parameters, and resolution level were considered, and tREX achieved consistently good performance in all settings. The results indicate that tREX is not only robust to resolution level, but it is also robust to model mis-specification and insensitive to parameter specifications. The comparisons are in terms of 3D structure estimation accuracy and preservation of topologically associated domains. However, tREX is slower computationally compared to the two optimization algorithms and PASTIS.

Application of tREX to the human lymphoblastoid cell line data on chromosomes 14 and 22 led to the construction of a 3D structure that is consistent with the FISH measurements of distances on several pairs of loci. It is shown that the corresponding distances predicted by tREX have higher correlation with the FISH measurements than any of the five comparison methods. Using the FISH measurements as the “gold standard”, this result indicates that tREX is a viable alternative to other existing methods.

Computational intensity is an important issue that deserves further discussion. As they are currently implemented, tREX, tPAM, BACH, and ChromSDE are all too computationally intensive to recover the 3D structure genome-wide in a single run. Nevertheless, compared among model-based approaches, tREX and tPAM are more apt for handling higher resolution data as well as inter-chromosomal data, as the sparse feature of the data helps with improving computational feasibility. PASTIS is a model-based approach that is computationally very efficient, but its performance appears to be unpredictable and unstable based on our observations from the simulation (there is large variability and extreme outliers; e.g. Additional file 1: Figure S1), and thus further evaluation is warranted. Ultimately, faster algorithms and further methodological innovations are needed for genome-wise 3D reconstruction, especially for higher resolution data. One idea is to use a subset of loci from each chromosome, “the anchors”, to build an overall architecture of the genome-wide 3D structure. Then a structure for each chromosome will be constructed and transformed to fit into the overall architecture, confirming to the positions of the anchors from the corresponding chromosome. Whether this strategy will work warrants further investigation, but it is out of the scope of the current paper.

## Methods

In this section, we describe the tREX model, the statistical inference from the model for estimating 3D structure, the design and execution of the in-silico study, the RMSD criterion for performance evaluation, and the standardization of distances to facilitate comparisons with the FISH measurement. Each of these topics will be discussed in turn in the following subsections.

### Truncated Random effect EXpression (tREX) model

Let *y*_*ij*_ be the count (an entry in the contact matrix) that represents the interaction intensity between loci *i* and *j*. For a set of *n* loci, their coordinates in the 3D space are denoted by $\boldsymbol {\omega } \equiv \{\vec {p}_{i}=({p^{x}_{i}},{p^{y}_{i}},{p^{z}_{i}}); \; i=1,\ldots,n\}$. We further use *d*_*ij*_ to denote the Euclidean distance between loci *i* and *j*:

$$d_{ij}=\sqrt{({p^{x}_{i}}-{p^{x}_{j}})^{2}+({p^{y}_{i}}-{p^{y}_{j}})^{2}+({p^{z}_{i}}-{p^{z}_{j}})^{2}}. $$

For reconstruction of 3D structure, the goal is to make inference on ***ω*** based on ***y***={*y*_*ij*_,1≤*i*<*j*≤*n*}, the data on the upper triangle of the 2D contact count matrix. Since *y*_*ij*_ represents count data, it has been modeled as from the Poisson distribution, that is, *Y*_*ij*_∼*Poisson*(*λ*_*ij*_) with intensity parameter *λ*_*ij*_, in the literature [13, 14, 16].

Note that the 2D data matrix is constructed for a given resolution. As resolution changes, the data matrix will change accordingly. For data at 1 Mb resolution, the proportion of zeros in intra-chromosomal data are minimal (<1 *%*). However, as resolution gets higher and higher, the percentage of zeros in the contact count matrix will get larger and larger. Large proportion of zeros is also observed for inter-chromosomal read count matrix when data from multiple chromosomes are analyzed together. Further, we would also like to point out that contact counts are correlated, but this nature of the data is being ignored in current methods that are model-based [12–14, 16].

To address the above features inherent in Hi-C data, we propose a truncated Random effect EXpression (tREX) model:

(1) $$\begin{array}{*{20}l} \log \lambda_{ij} = \beta_{0}+ \beta_{1} \log d_{ij}+\boldsymbol{z}^{T}_{ij}\boldsymbol{\gamma}+W_{ij}, \end{array} $$

with the likelihood of ***y*** for $\mathcal {I}\equiv \{(i,j);y_{\textit {ij}} \neq 0\}$ (i.e. excluding zero counts) being

(2) $$\begin{array}{*{20}l} \log p(\boldsymbol{y}; \boldsymbol{\theta}, \boldsymbol{\omega}) \propto \mathop{\sum\sum}_{(i,j)\in {\mathcal{I}}} \left\{y_{ij}\log \lambda_{ij}-\log(e^{\lambda_{ij}}-1)\right\}. \end{array} $$

In Eq. (1), *λ*_*ij*_ is the interaction intensity between loci *i* and *j* as defined earlier; *d*_*ij*_ is the Euclidean distance between *i* and *j*, also as defined earlier; $\boldsymbol {z}^{T}_{\textit {ij}}$ is a vector of covariates (e.g. fragment length, GC content, and mappability score) to address systematic biases (acting as normalization of the data); *W*_*ij*_ is the random effect that will be discussed more below; *β*_0_,*β*_1_, and ***γ*** are the coefficients (effect sizes) of the corresponding factors. Note that the restriction of *β*_1_<0 is imposed to reflect the biological property that two loci in close proximity in the 3D space is likely to interact more. In Eq. (2), ***θ*** denotes the collection of all model parameters, and ***ω*** is the collection of the coordinates of the 3D structure. It is noteworthy that the number of summations necessary for the evaluation of the log-likelihood (2) is decreased for a data matrix with many zeros. This can lead to significant reduction in computational time.

We can see that the truncated Poisson Architecture Model (tPAM), as detailed in the Additional file 1: S1, is a special case of tREX. The addition of the random effect *W*_*ij*_ to the tREX model for the intensity parameter of the truncated Poisson is a nontrivial generalization that leads to two very important properties. First, in tPAM, all frequencies {*y*_*ij*_} in the contact matrix are assumed to be independent, thus ignoring the dependency inherent in the data for pairs of contacts that share a common locus. On the other hand, tREX takes such dependency into account through the inclusion of the random effect component *W*_*ij*_. This effectively induces correlation between the mean contact frequencies of two pairs that share a common locus. That is, the mean values of contact frequencies are no longer fixed values; they are in fact random variables with potentially non-zero correlations (See Additional file 1: S2 for a detailed derivation). Further, a Poisson model may not fit the data well as it cannot accommodate overdispersion that is typically seen in sequencing data. More specifically, we can see that the mean of a truncated Poisson distribution is in fact larger than its variance (Additional file 1: S1). By introducing the random effect component, tREX can accommodate data with a greater variability. Details are provided in Additional file 1: S3, which shows that the variance of a contact frequency can be greater than its mean under the tREX model.

### Inference

Model (1) suffers from non-identifiability because the estimated 3D structure $\hat {\boldsymbol {\omega }}$ is not invariant to scale, rotation, reflection, and translation [16]. To resolve this issue, without loss of generality, we can fix *β*_0_ to be an arbitrarily predefined quantity. Note that *β*_0_ controls the scale of the 3D structure; thus fixing *β*_0_ will effectively lead to the structure being estimated only up to a scale. However, this is not an issue since the relative distance does not affect the predicted structure and its correlation with genomic functions [9]. Following [16], we further place the following restrictions on ***ω*** to make it estimable, as four conditions on the structure are sufficient to uniquely determine the 3D structure: $\vec {p}_{1}=(0,0,0)$, $\vec {p}_{2}=({p_{2}^{x}},0,{p_{2}^{z}})$ with ${p_{2}^{z}}>0$, $\vec {p}_{3}=({p_{3}^{x}},{p_{3}^{y}},{p_{3}^{z}})$ with ${p_{3}^{y}}>0$, and $\vec {p}_{n}=({p_{n}^{x}},0,0)$ with ${p_{n}^{x}}>0$. Further, to accommodate the estimable conditions imposed on ***ω*** in the course of inference, we consider an isometric transformation *I*, which is compositions of translation, rotations, and reflection. Details can be found in [16]. To sample from the posterior distributions of ***θ***, we use Metropolis-Hastings algorithms and in particular the Gibbs sampler whenever the conditional distribution of a parameter is of a commonly known one. In sampling the posterior of ***ω***, we employed Hamiltonian MCMC to handle more effectively the high correlations among the samples [18].

### Design, simulation, and analysis of data in the in-silico study

The structure we used as the “gold standard”, ***ω***_*gs*_, is one used in the literature already [16]. We selected this structure because it was estimated from real Hi-C data. Further, it depicts two topological domains [15] so that there are two clear substructures within the overall structure (Fig. 3(a)). With this feature of the underlying structure, it is feasible to study the ability of a method for detecting the domains and to assess the impact of resolution on each method.

#### Two models for simulating data

Recall that higher resolution data will lead to sparser 2D contact matrix, that is, higher percentage of zeros in the entries. As such, we design our in-silico study to assess the impact of resolution on the performance of the methods by simulating from a zero-inflated Poisson model to mimic different levels of resolution in Hi-C data:

(3) $$\begin{array}{@{}rcl@{}} P(Y_{ij}=0) & = & \pi +(1-\pi)e^{-\lambda_{ij}}, \\ P(Y_{ij}=y_{ij}) & = & (1-\pi)\frac{\lambda_{ij}^{y_{ij}}e^{-\lambda_{ij}}}{y_{ij}!}, \; y_{ij}=1, 2, \cdots, \end{array} $$

where *i,j*=1,2,⋯,43 and *i*<*j* as ***ω***_*gs*_ is a structure with 43 loci, and *π* is the mixing proportion used to represent data resolution. From the human lymphoblastoid Hi-C intra-chromosomal data [1], percentages of zero counts for 1 Mb, 50, 25, 20, and 10 Kb are approximately 0% (<1 *%*), 10, 20, 30, and 60 %, respectively. Therefore, we use these five different percentages as a surrogate for representing the different levels of resolution, and these two terms, level of resolution and percentage of zeros, are used interchangeably in this article. The specification of the mean interaction intensity, *λ*_*ij*_, leads to two different models. For each of the model and a resolution, 50 data sets are simulated.

##### *NRE*

The first model we consider is the No Random Effect (NRE) model:

$$ \begin{aligned} \log \lambda_{ij}& = \beta_{0} + \beta_{1} \log d_{ij}+\gamma_{1}\log (z_{l,i}z_{l,j})\\ &\quad+\gamma_{2}\log (z_{g,i} z_{g,j})+\log (z_{m,i} z_{m,j}), \end{aligned} $$

where the coefficient *β*1 was set to be −0.434, which was the estimate obtained along with the gold standard structure ***ω***_*gs*_ [16]. Note that this parameter is far from −1 used in many optimization-based methods. For (*γ*_1_,*γ*_2_), three sets of values are considered to entertain a variety of potential covariates: (0.3,0.3),(0.05,0.25), and (0.05,−0.25). Following the argument in [9], we set *β*_0_=3 arbitrarily as its value only affects the scale of the predicted structure, thus not altering its correlation with genomic functions. Finally, to mimic real Hi-C data and guided by the ranges of data for three factors that can affect the counts [1], we set *z*_*l,i*_∼*Unif*(0.2,0.3), *z*_*g,i*_∼*Unif*(0.4,0.5) and *z*_*m,i*_∼*Unif*(0.9,1), where *Unif*(.) denotes a uniform distribution. Note that this model coincides with the analysis model employed by tPAM, BACH, and PASTIS, and therefore these methods are expected to perform well, especially when there is no zero-inflation.

##### *ST*

In real Hi-C data, the contact counts are correlated. To investigate the performance of the methods in the presence of dependency between *y*_*ij*_, we also consider the model

$$ \begin{aligned} \log \lambda_{ij} &= \beta_{0} + \beta_{1} \log d_{ij}+\gamma_{1}\log (z_{l,i}z_{l,j})+\gamma_{2}\log (z_{g,i} z_{g,j})\\ &\quad+\log (z_{m,i} z_{m,j}) + W_{ij}, \end{aligned} $$

where we set *W*_*ij*_=*X*_*i*_+*X*_*j*_+*U*_*ij*_, and we assume *X*_*i*_,*i*=1,2,⋯*n* to be independently distributed as skewed-t: *ST*(0,scale = 0.3,shape = 1,df = 10), and $U_{\textit {ij}} \stackrel {iid}{\sim } N(0,{\sigma _{u}^{2}})$ for all pairs of *i* and *j*. This model is referred to as the ST model. The other parameters are set as in the NRE model; in particular, three sets of values for (*γ*_1_,*γ*_2_) are considered. In the tREX analysis model, on the other hand, *W*_*ij*_ is treated as normally distributed (which is also assumed in Additional file 1: S2 and 3). Thus, the analysis model is different from the simulation, which provides a means to study robustness to model mis-specification for tREX and the other model-based methods.

#### Analysis and RMSD evaluation criterion

We compared tREX with its fellow modeling-based methods (BACH [13], tPAM [16] and PASTIS [14]) as well as optimization-based methods (ShRec3D [10] and ChromSDE [9]). In fitting tREX, tPAM, and BACH to the simulation data, we normalized the data by incorporating the systematic bias information as covariates to the model. In contrast, we first normalized the data by HiCNorm [19] before using ShRec3D, ChromSDE and PASTIS to the normalized data. In order to assess and compare estimation accuracy of the methods, we need to take into account the fact that the estimated architecture is accurate only up to a scaling factor. Therefore, we first estimate the scaling factor *α* by the least squares model as

$$\hat{\alpha} ={\arg\min_{\alpha}} \left\{\mathop{\sum\sum}_{1\leq i<j \leq n}\left(d_{ij}-\alpha \hat{d}_{ij}\right)^{2} \right\}. $$

Then we employ the root mean square deviation (RMSD) to compare the estimated 3D coordinates with the underlying 3D coordinates:

$$RMSD = \sqrt{\frac{1}{n}\sum_{i=1}^{n} \{\Im(\hat{\alpha}\hat{p}_{i})-\Im(p_{i})\}^{2}}, $$

where *I* is the composition of isometric transformations as described above. Note that in Figs. 1 and 2, the RMSD plotted is in fact $\sqrt {n}$RMSD to make it easier to label the y-axis.

### Standardization of distances

To evaluate the performance for tREX and the other methods on the real data, we compared the estimates of the pairwise distances to those of FISH, the gold standard measurements. To facilitate such a comparison due to scale differences, we first calculated a unit-less distance $\tilde {d}(L_{i},L_{j})$ by dividing each distance *d*(*L*_*i*_,*L*_*j*_) by the median distance between *L*_3_ and *L*_4_ (the largest median distance among all pairs from FISH). Note that the median is taken over 100 measurements for FISH and 10,000 estimates for tREX, tPAM, and BACH. For PASTIS, ShRec3D and ChromSDE, there is only a single consensus estimate.

## Additional file

Additional file 1 **Web-based supplementary materials for “impact of data resolution on three-dimensional structure inference methods”.** (PDF 234 kb)
