# Supplementary material for: Impact of data resolution on three-dimensional structure inference methods
Source: BMC Bioinformatics. 2016 Feb 6;17:70. doi: 10.1186/s12859-016-0894-z (PMC4744395; doi:10.1186/s12859-016-0894-z)
Supplement: Supplementary file 1 — Web-based supplementary materials for “impact of data resolution on three-dimensional structure inference methods”. (PDF 234 kb) [file 12859_2016_894_MOESM1_ESM.pdf]

Web-Based Supplementary Materials for "Impact of Data Resolution on Three-Dimensional Structure Inference Methods"

Jincheol Park[1,2,3], Shili Lin[2,3]

[1]Department of Statistics, Keimyung University,

[2]Department of Statistics, [3]Mathematical Biosciences Institute

The Ohio State University, Columbus, OH 43210

S1. Truncated Poisson Architecture Model (tPAM)

The setup of tPAM unfolds as in tREX, but it assumes that the interaction counts are independent and does not allowed for over-dispersion, as we elaborate below.

Let y_{ij} be the count (an entry in the contact matrix) that represents the interaction intensity between loci i and j. For a set of n loci, their coordinates in the 3D space are denoted by $\boldsymbol{\omega} \equiv \{\vec{p}_i = (p_i^x, p_i^y, p_i^z);\ i = 1, \ldots, n\}$. We further use d_{ij} to denote the Euclidean distance between loci i and j:

$$d_{ij} = \sqrt{(p_i^x - p_j^x)^2 + (p_i^y - p_j^y)^2 + (p_i^z - p_j^z)^2}.$$

For reconstruction of 3D structure, tPAM assumes that y_{ij}, being count data, follows the truncated Poisson distribution with intensity parameter λ_{ij}, which is modeled as follows:

$$\log \lambda_{ij} = \beta_0 + \beta_1 \log d_{ij} + \boldsymbol{z}_{ij}^T \boldsymbol{\gamma}, \tag{1}$$

where d_{ij} is the Euclidean distance between i and j; $\boldsymbol{z}_{ij}^T$ is a vector of covariates (e.g. fragment length, GC content, and mappability score) to address systematic biases (acting as normalization of the data); β_0, β_1, and $\boldsymbol{\gamma}$ are the coefficients (effect sizes) of the corresponding factors.

This model differs from the tREX model proposed in this paper in the omission of the random component W_{ij} (equation (1) of main paper). Lacking this term has two major drawbacks. First, in forming the likelihood of tPAM by multiplying the probabilities over all (i, j) cells (but excluding those with 0 contact counts) of the 2D contact matrix, we are essentially assuming that y_{ij} is independent of $y_{i'j}$ for $i \neq i'$, thus ignoring the dependency inherent by the virtue of these two counts sharing a common locus j.

The second drawback is tPAM's inability to accommodate potential over-dispersion of sequencing data. More specifically, the mean and the variance of a truncated Poisson random variable Y with parameter λ are

$$\begin{aligned} E(Y) &= \frac{\lambda e^{\lambda}}{e^{\lambda}-1}, \\ Var(Y) &= E(Y) - \frac{\lambda^2 e^{\lambda}}{(e^{\lambda}-1)^2}. \end{aligned}$$

As such, one can see that the variance is in fact smaller than the mean for a truncated Poisson distribution, leading to the opposite effect of what one would desire (having larger variance than the mean to accommodate over-dispersion). The proposed tREX is to address these two main issues, we we elaborate in the following two sections.

S2. Dependence of mean contact counts for tREX

Recall that in tREX, the observed count y_{ij} also follows a truncated Poisson distribution but with its intensity parameter modeled as

$$\log \lambda_{ij} = \beta_0 + \beta_1 \log d_{ij} + \boldsymbol{z}_{ij}^T \boldsymbol{\gamma} + W_{ij}, \quad (2)$$

where the variables and parameters in $\beta_0 + \beta_1 \log d_{ij} + \boldsymbol{z}_{ij}^T \boldsymbol{\gamma}$ are as explained in S1, and we assume $W_{ij} \equiv X_i + X_j + U_{ij}$ where $X_i \overset{iid}{\sim} N(0, \sigma_x^2)$, for $i = 1, \cdots, n$, $U_{ij} \overset{iid}{\sim} N(0, \sigma_u^2)$, for $i, j = 1, \cdots, n, i \neq j$, and $\{X_i, i = 1, \cdots, n\}$ and $\{U_{ij}, i, j = 1, \cdots, n, i \neq j\}$ are independent.

Let $i \neq i'$. Then

$$
\begin{aligned}
Cov(Y_{ij}, Y_{i'j'}) &= E(Y_{ij}Y_{i'j'}) - E(Y_{ij})E(Y_{i'j'}) \\
&= E\{E(Y_{ij}Y_{i'j'}|\lambda_{ij}, \lambda_{i'j'})\} - E\{E(Y_{ij}|\lambda_{ij})\}E\{E(Y_{i'j'}|\lambda_{i'j'})\} \\
&= E\left(\frac{e^{\lambda_{ij}}}{e^{\lambda_{ij}}-1}\frac{e^{\lambda_{i'j'}}}{e^{\lambda_{i'j'}}-1}\lambda_{ij}\lambda_{i'j'}\right) - E\left(\frac{e^{\lambda_{ij}}}{e^{\lambda_{ij}}-1}\lambda_{ij}\right)E\left(\frac{e^{\lambda_{i'j'}}}{e^{\lambda_{i'j'}}-1}\lambda_{i'j'}\right) \\
&= Cov\left(\frac{e^{\lambda_{ij}}}{e^{\lambda_{ij}}-1}\lambda_{ij}, \frac{e^{\lambda_{i'j'}}}{e^{\lambda_{i'j'}}-1}\lambda_{i'j'}\right).
\end{aligned}
$$

Now note that random variable $Z_{ij} = \lambda_{ij}e^{\lambda_{ij}}/(e^{\lambda_{ij}} - 1)$ is a function of random variable W_{ij}, that is, $Z = f(W_{ij})$, where f is a function whose inverse exists. Similarly, $Z_{i'j'} = \lambda_{i'j'}e^{\lambda_{i'j'}}/(e^{\lambda_{i'j'}} - 1) = g(W_{i'j'})$. Furthermore,

$$
Cov(W_{ij}, W_{i'j'}) = \begin{cases} \sigma_x^2 & \text{if } j = j'; \\ 0 & \text{if } j \neq j'. \end{cases} \tag{3}
$$

Since the W_{ij}'s are normally distributed, we can see that, for two pairs that do not share a common locus (that is, $j \neq j'$), they are not only uncorrelated, they are in fact independent. On the other hand, if two pairs do share a common locus (that is, $j = j'$),

$$
Cov\left(\frac{e^{\lambda_{ij}}}{e^{\lambda_{ij}}-1}\lambda_{ij}, \frac{e^{\lambda_{i'j'}}}{e^{\lambda_{i'j'}}-1}\lambda_{i'j'}\right) \neq 0,
$$

because otherwise W_{ij} and $W_{i'j'}$ would have to be independent, which we have already shown not to be the case (equation (3)). Therefore, for two pairs of loci sharing a common locus, the means of their observed contact counts are dependent.

S3. Accommodation of over-dispersion with tREX

As we see in S1, for the tPAM model, $E(Y_{ij}) < Var(Y_{ij})$, that is, the mean is in fact smaller than the variance. As such, tPAM fails to account for over-dispersion typically seen in

sequencing data. On the other hand, as we derive in the following, the tREX model can lead to $Var(Y_{ij}) > E(Y_{ij})$, hence its ability to accommodate over-dispersion. For simplicity without ambiguity, we drop the subscript ij in Y_{ij} and λ_{ij} here after. We first write out the variance as follows:

$$\begin{aligned} Var(Y) &= E\left\{Var\left(Y|\lambda\right)\right\} + Var\left\{E\left(Y|\lambda\right)\right\} \\ &= E\left\{E\left(Y|\lambda\right) - \frac{\lambda^2 e^\lambda}{\left(e^\lambda - 1\right)^2}\right\} + Var\left(\frac{\lambda e^\lambda}{e^\lambda - 1}\right) \\ &= E\left(Y\right) + Var\left(\frac{\lambda e^\lambda}{e^\lambda - 1}\right) - E\left\{\frac{\lambda^2 e^\lambda}{\left(e^\lambda - 1\right)^2}\right\}. \end{aligned}$$

We let

$$\mathcal{A} = Var\left(\frac{\lambda e^\lambda}{e^\lambda - 1}\right) - E\left\{\frac{\lambda^2 e^\lambda}{\left(e^\lambda - 1\right)^2}\right\}.$$

Then $\mathcal{A} > 0$ implies that $Var(Y) > E(Y)$. It follows from

$$Var\left(\frac{\lambda e^\lambda}{e^\lambda - 1}\right) = E\left\{\frac{\lambda^2 e^{2\lambda}}{\left(e^\lambda - 1\right)^2}\right\} - E^2\left(\frac{\lambda e^\lambda}{e^\lambda - 1}\right)$$

that a lower bound of $\mathcal{A}$ can be obtained

$$\begin{aligned} \mathcal{A} &= E\left\{\frac{\lambda^2 e^{2\lambda}}{\left(e^\lambda - 1\right)^2}\right\} - E^2\left(\frac{\lambda e^\lambda}{e^\lambda - 1}\right) - E\left\{\frac{\lambda^2 e^\lambda}{\left(e^\lambda - 1\right)^2}\right\} \\ &= E\left(\frac{\lambda^2 e^\lambda}{e^\lambda - 1}\right) - E^2\left(\frac{\lambda e^\lambda}{e^\lambda - 1}\right) \\ &> Var(\lambda) + E\left(\frac{\lambda^2}{e^\lambda - 1}\right) - 2E\left(\lambda\right) - 1 \qquad (4) \\ &> Var(\lambda) - 2E\left(\lambda\right) - 1, \qquad (5) \end{aligned}$$

where inequality (4) is obtained because

$$E\left(\frac{\lambda^2 e^\lambda}{e^\lambda - 1}\right) = E\left(\lambda^2\right) + E\left(\frac{\lambda^2}{e^\lambda - 1}\right),$$

$$E\left(\frac{\lambda e^\lambda}{e^\lambda - 1}\right) = E\left(\lambda\right) + E\left(\frac{\lambda}{e^\lambda - 1}\right), \quad \text{and}$$

$$E\left(\frac{\lambda}{e^\lambda - 1}\right) < 1 \quad \text{since } 0 < \frac{\lambda}{e^\lambda - 1} < 1.$$

On the other hand, inequality (5) is obtained simply noting that $E(\lambda^2/(e^\lambda - 1)) > 0$.

Making the same assumption as in S2 for W, it can be seen that λ follows a log-normal distribution with the mean and the variance of $\log \lambda$ being $\mu = \beta_0 + \beta_1 \log d_{ij} + \boldsymbol{z}_{ij}^T \boldsymbol{\gamma}$ and $\sigma^2 = 2\sigma_x^2 + \sigma_u^2$, respectively. Then one has

$$E\left(\lambda\right) = e^{\mu + \sigma^2/2}, \quad and$$

$$Var\left(\lambda\right) = \left(e^{\sigma^2} - 1\right) e^{2\mu + \sigma^2}.$$

Hence, (5) (a lower bound of $\mathcal{A}$) can be rewritten as

$$Var(\lambda) - 2E\left(\lambda\right) - 1 = (e^{\sigma^2} - 1)e^{2\mu + \sigma^2} - 2e^{\mu + \sigma^2/2} - 1.$$

Setting the lower bound given above to be positive, we can solve the inequality to obtain the following relationship between μ and σ^2 by completing the square:

$$\mu > h(\sigma^2) = \log\left(\frac{1 + e^{\sigma^2/2}}{e^{\sigma^2} - 1}\right) - \sigma^2/2.$$

Note that $dh(\sigma^2)/d\sigma^2 < 0$, and thus $h(\sigma^2)$ is a decreasing function of $\sigma^2 = 2\sigma_x^2 + \sigma_u^2$. Therefore, by letting the variance in the random effect component W be sufficiently large, one can accommodate the over-dispersion problem in sequencing data with tREX. That is, it is completely within the capability of tREX to accommodate over-dispersion if such a feature indeed exists in the data.

Supplementary Table 1: P-values of Wilcoxon signed-rank tests comparing the performance of tREX with each of the comparison methods for the model with parameters $(\beta_0, \beta_1, \gamma_1, \gamma_2) = (3, -0.434, 0.05, -0.25)$. The results for the NRE model are given in the top segment (A) and those for the ST model are given in the bottom segment (B).

(A). NRE Model

		Resolution/Percent Zeros				
Criterion	Method	0%	10%	20%	30%	60%
RMSD	tPAM	6.4×10^{-1}	8.2×10^{-1}	9.9×10^{-1}	9.9×10^{-1}	9.5×10^{-1}
	BACH	3.1×10^{-1}	1.8×10^{-8}	1.4×10^{-9}	8.0×10^{-10}	4.6×10^{-10}
	PASTIS	4.1×10^{-10}	2.9×10^{-1}	9.9×10^{-1}	9.6×10^{-9}	1.9×10^{-7}
	ShRec3D	4.1×10^{-10}	1.7×10^{-15}	1.7×10^{-15}	1.7×10^{-15}	1.1×10^{-9}
	ChromSDE	4.8×10^{-4}	3.1×10^{-2}	5.3×10^{-2}	2.5×10^{-2}	7.1×10^{-3}
Correlation	tPAM	1.4×10^{-1}	4.7×10^{-1}	9.5×10^{-1}	9.9×10^{-1}	4.8×10^{-1}
	BACH	1.6×10^{-1}	1.8×10^{-8}	5.9×10^{-10}	3.8×10^{-10}	3.8×10^{-10}
	PASTIS	4.6×10^{-10}	9.9×10^{-1}	9.9×10^{-1}	5.9×10^{-10}	4.3×10^{-10}
	ShRec3D	3.8×10^{-10}	1.3×10^{-11}	4.3×10^{-10}	2.5×10^{-9}	3.9×10^{-7}
	ChromSDE	7.3×10^{-1}	9.7×10^{-1}	8.7×10^{-1}	9.9×10^{-1}	5.5×10^{-1}

(B). ST Model

		Resolution/Percent Zeros				
Criterion	Method	0%	10%	20%	30%	60%
RMSD	tPAM	4.8×10^{-5}	4.2×10^{-6}	6.6×10^{-5}	3.1×10^{-4}	6.4×10^{-6}
	BACH	9.3×10^{-5}	5.1×10^{-7}	7.9×10^{-6}	4.0×10^{-5}	3.8×10^{-7}
	PASTIS	5.6×10^{-6}	4.0×10^{-2}	1.5×10^{-4}	9.8×10^{-1}	3.7×10^{-1}
	ShRec3D	7.9×10^{-3}	8.5×10^{-4}	1.2×10^{-2}	1.0×10^{-1}	1.9×10^{-3}
	ChromSDE	9.0×10^{-3}	7.7×10^{-5}	9.7×10^{-3}	3.7×10^{-2}	3.5×10^{-3}
Correlation	tPAM	1.3×10^{-9}	4.1×10^{-10}	5.8×10^{-9}	1.9×10^{-8}	4.1×10^{-10}
	BACH	2.7×10^{-9}	6.7×10^{-10}	4.1×10^{-10}	5.2×10^{-10}	3.8×10^{-10}
	PASTIS	7.1×10^{-10}	6.1×10^{-6}	6.4×10^{-8}	8.0×10^{-1}	1.2×10^{-1}
	ShRec3D	2.5×10^{-7}	3.6×10^{-8}	2.4×10^{-3}	8.4×10^{-4}	6.7×10^{-9}
	ChromSDE	2.3×10^{-9}	1.0×10^{-9}	2.2×10^{-8}	1.2×10^{-7}	8.0×10^{-10}

Supplementary Table 2: P-values of Wilcoxon signed-rank tests comparing the performance of tREX with each of the comparison methods for the model with parameters $(\beta_0, \beta_1, \gamma_1, \gamma_2) = (3, -0.434, 0.05, 0.25)$. The results for the NRE model are given in the top segment (A) and those for the ST model are given in the bottom segment (B).

(A). NRE Model

		Resolution/Percent Zeros				
Criterion	Method	0%	10%	20%	30%	60%
RMSD	tPAM	1.9×10^{-1}	2.2×10^{-1}	2.3×10^{-1}	8.1×10^{-1}	2.3×10^{-2}
	BACH	3.1×10^{-2}	8.6×10^{-9}	7.1×10^{-10}	3.8×10^{-10}	4.1×10^{-10}
	PASTIS	3.8×10^{-10}	4.1×10^{-2}	9.7×10^{-1}	9.9×10^{-1}	9.9×10^{-1}
	ShRec3D	3.8×10^{-10}	4.1×10^{-10}	3.8×10^{-10}	4.1×10^{-10}	8.5×10^{-10}
	ChromSDE	2.7×10^{-7}	8.3×10^{-5}	8.3×10^{-3}	3.7×10^{-3}	1.1×10^{-6}
Correlation	tPAM	5.4×10^{-3}	3.4×10^{-3}	4.9×10^{-1}	1.8×10^{-1}	1.2×10^{-2}
	BACH	2.3×10^{-2}	4.6×10^{-10}	3.8×10^{-10}	3.8×10^{-10}	3.8×10^{-10}
	PASTIS	5.9×10^{-10}	9.4×10^{-1}	9.9×10^{-1}	9.9×10^{-1}	9.9×10^{-1}
	ShRec3D	4.1×10^{-10}	3.8×10^{-10}	4.1×10^{-10}	1.5×10^{-9}	2.4×10^{-7}
	ChromSDE	1.6×10^{-3}	1.6×10^{-1}	4.2×10^{-1}	3.5×10^{-1}	8.0×10^{-2}

(B). ST Model

		Resolution/Percent Zeros				
Criterion	Method	0%	10%	20%	30%	60%
RMSD	tPAM	6.1×10^{-4}	1.2×10^{-5}	2.2×10^{-5}	2.1×10^{-4}	1.0×10^{-4}
	BACH	2.3×10^{-4}	1.9×10^{-6}	3.7×10^{-6}	2.0×10^{-6}	1.1×10^{-8}
	PASTIS	2.0×10^{-6}	4.2×10^{-2}	6.3×10^{-1}	5.8×10^{-1}	07.5×10^{-1}
	ShRec3D	7.6×10^{-3}	1.0×10^{-3}	2.7×10^{-2}	2.7×10^{-3}	1.7×10^{-3}
	ChromSDE	7.4×10^{-3}	1.4×10^{-4}	5.7×10^{-3}	1.2×10^{-4}	4.6×10^{-4}
Correlation	tPAM	1.2×10^{-9}	4.3×10^{-10}	5.6×10^{-10}	6.8×10^{-9}	4.3×10^{-10}
	BACH	3.1×10^{-9}	4.3×10^{-10}	3.8×10^{-10}	4.1×10^{-10}	3.8×10^{-10}
	PASTIS	6.7×10^{-10}	3.5×10^{-6}	7.4×10^{-3}	3.5×10^{-1}	1.2×10^{-2}
	ShRec3D	3.3×10^{-6}	2.4×10^{-9}	1.7×10^{-7}	6.5×10^{-4}	1.6×10^{-10}
	ChromSDE	2.4×10^{-9}	3.1×10^{-9}	4.9×10^{-10}	2.3×10^{-8}	4.9×10^{-10}

Supplementary Table 3: Average silhouette width ratio for the model with parameters $(\beta_0, \beta_1, \gamma_1, \gamma_2) = (3, -.434, 0.05, -0.25)$. Each number represents the ratio of the average silhouette width of the estimated structure and the average silhouette width of the underlying 3D structure.

		Resolution				
Model	Method	0%	10%	20%	30%	60%
NRE	tREX	0.854	0.830	0.816	0.798	0.738
	tPAM	0.845	0.830	0.826	0.816	0.744
	BACH	0.843	0.743	0.673	0.599	0.409
	PASTIS	0.754	0.875	0.911	0.931	0.884
	ShRec3D	0.738	0.734	0.729	0.723	0.683
	ChromSDE	0.864	0.854	0.839	0.822	0.738
ST	tREX	0.742	0.726	0.703	0.689	0.634
	tPAM	0.610	0.596	0.590	0.572	0.520
	BACH	0.608	0.574	0.533	0.494	0.364
	PASTIS	0.601	0.690	0.601	0.721	0.635
	ShRec3D	0.673	0.668	0.656	0.648	0.576
	ChromSDE	0.653	0.646	0.630	0.616	0.549

Supplementary Table 4: Average silhouette width ratio for the model with parameters $(\beta_0, \beta_1, \gamma_1, \gamma_2) = (3, -.434, 0.05, 0.25)$. Each number represents the ratio of the average silhouette width of the estimated structure and the average silhouette width of the underlying 3D structure.

		Resolution				
Model	Method	0%	10%	20%	30%	60%
NRE	tREX	0.848	0.839	0.822	0.799	0.752
	tPAM	0.846	0.830	0.830	0.803	0.736
	BACH	0.846	0.751	0.667	0.599	0.406
	PASTIS	0.754	0.876	0.904	0.935	0.887
	ShRec3D	0.736	0.733	0.728	0.722	0.683
	ChromSDE	0.860	0.850	0.842	0.818	0.734
ST	tREX	0.740	0.732	0.712	0.692	0.651
	tPAM	0.602	0.603	0.588	0.573	0.520
	BACH	0.616	0.565	0.531	0.491	0.364
	PASTIS	0.601	0.693	0.715	0.721	0.649
	ShRec3D	0.673	0.667	0.658	0.647	0.576
	ChromSDE	0.653	0.646	0.629	0.616	0.553

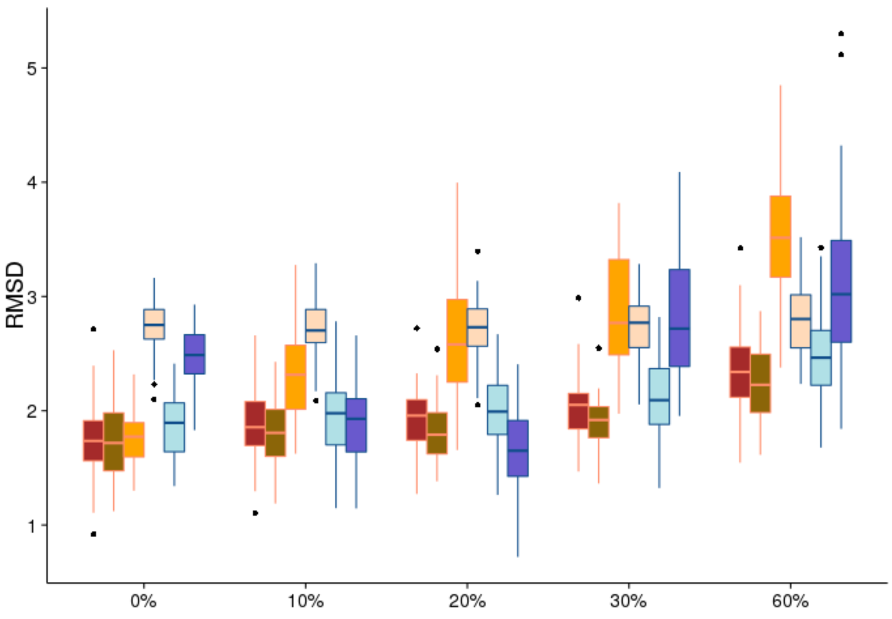

(a)

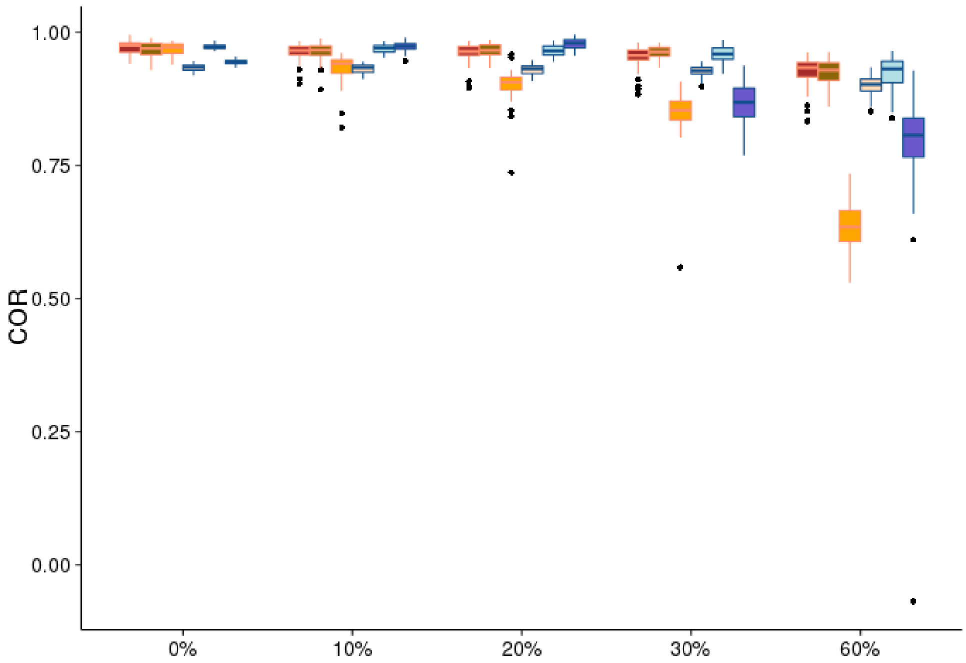

(b)

Supplementary Figure S1: Boxplots for comparing 3D estimation accuracy of six methods under NRE model and parameter setting $(\beta_0, \beta_1, \gamma_1, \gamma_2) = (3, -0.434, 0.05, -0.25)$. The comparison are for data simulated from the NRE model based on two criteria: (a) RMS D, and (b) Correlation. For each resolution/percent zeros, the six boxplots are for tREX, tPAM, BACH, ShRec3D, ChromSDE, and PASTIS, in that order.

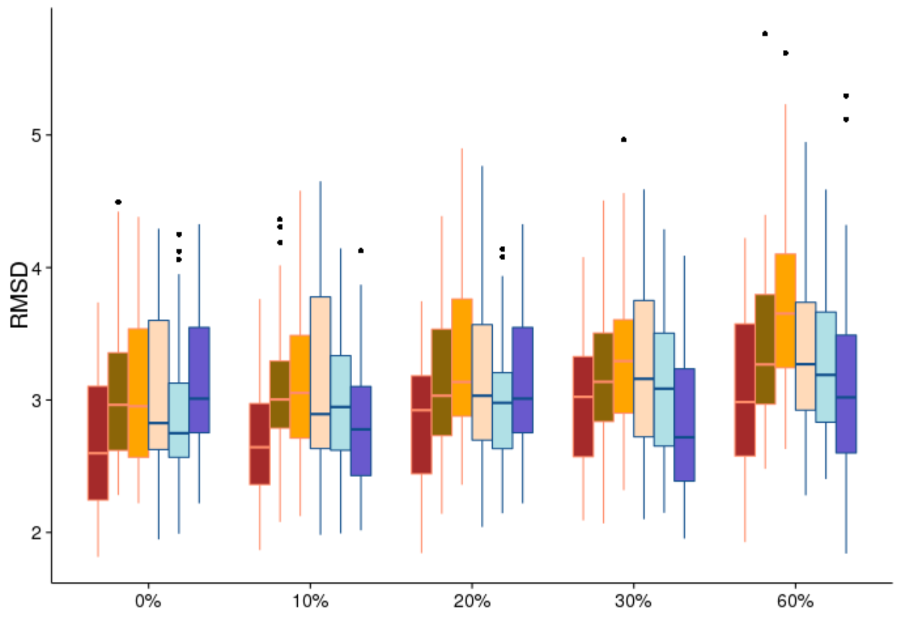

(a)

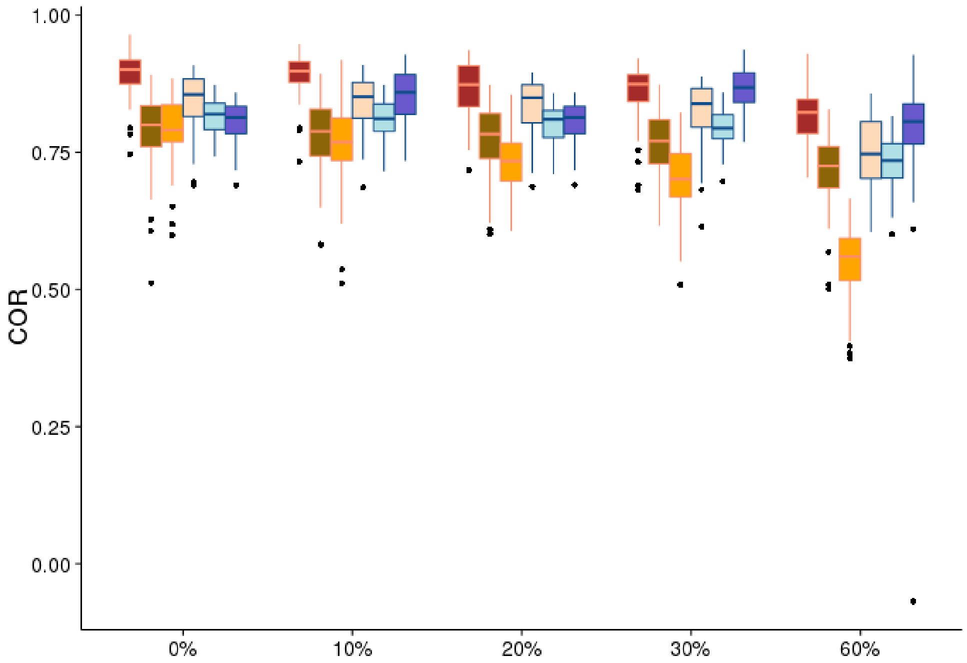

(b)

Supplementary Figure S2: Boxplots for comparing 3D estimation accuracy of six methods under ST model and parameter setting $(\beta_0, \beta_1, \gamma_1, \gamma_2) = (3, -0.434, 0.05, -0.25)$. The comparison are for data simulated from the NRE model based on two criteria: (a) RMS D, and (b) Correlation. For each resolution/percent zeros, the six boxplots are for tREX, tPAM, BACH, ShRec3D, ChromSDE, and PASTIS, in that order.

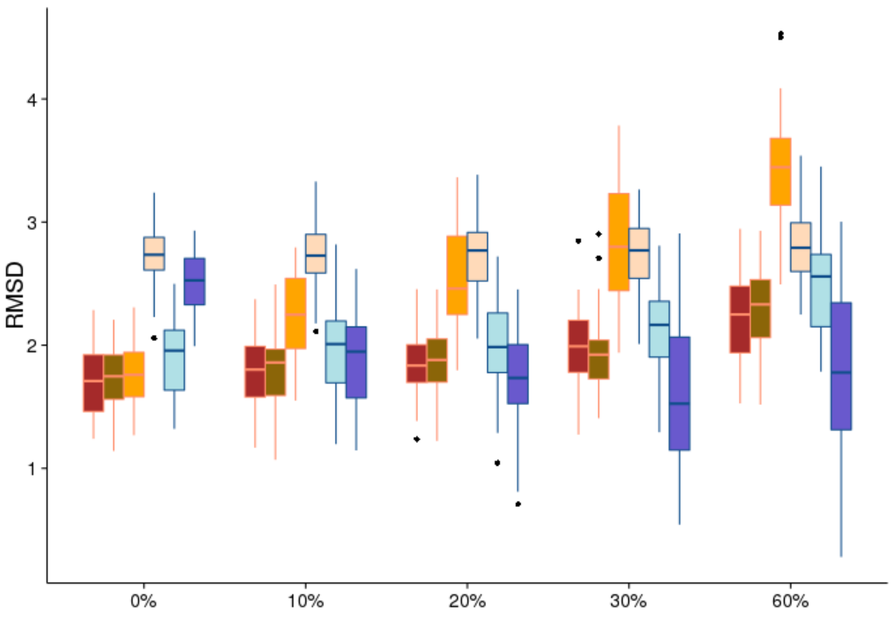

(a)

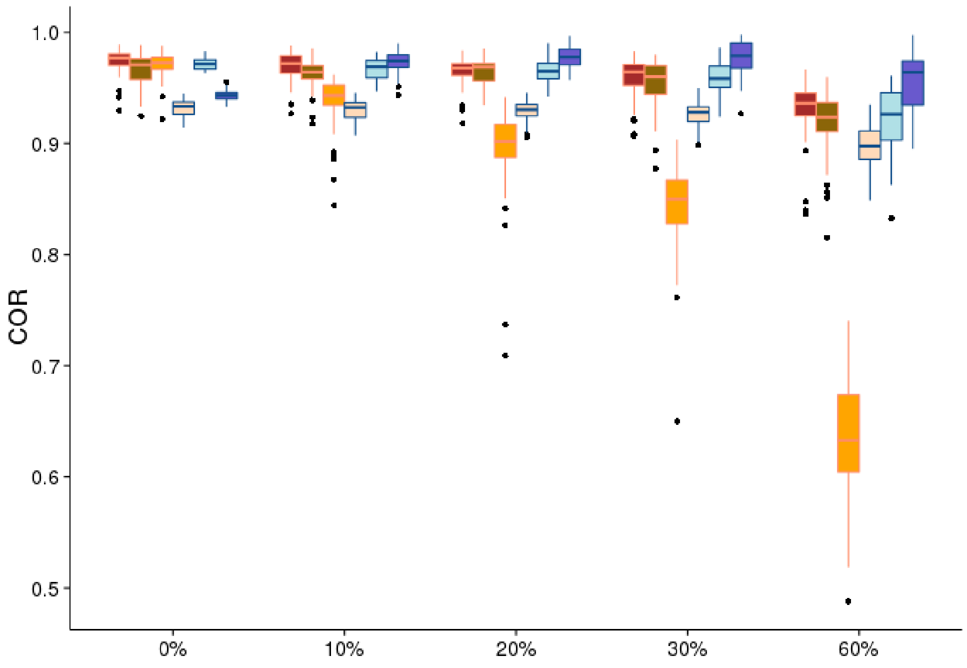

(b)

Supplementary Figure S3: Boxplots for comparing 3D estimation accuracy of six methods under NRE model and parameter setting $(\beta_0, \beta_1, \gamma_1, \gamma_2) = (3, -0.434, 0.05, 0.25)$. The comparison are for data simulated from the NRE model based on two criteria: (a) RMS D, and (b) Correlation. For each resolution/percent zeros, the six boxplots are for tREX, tPAM, BACH, ShRec3D, ChromSDE, and PASTIS, in that order.

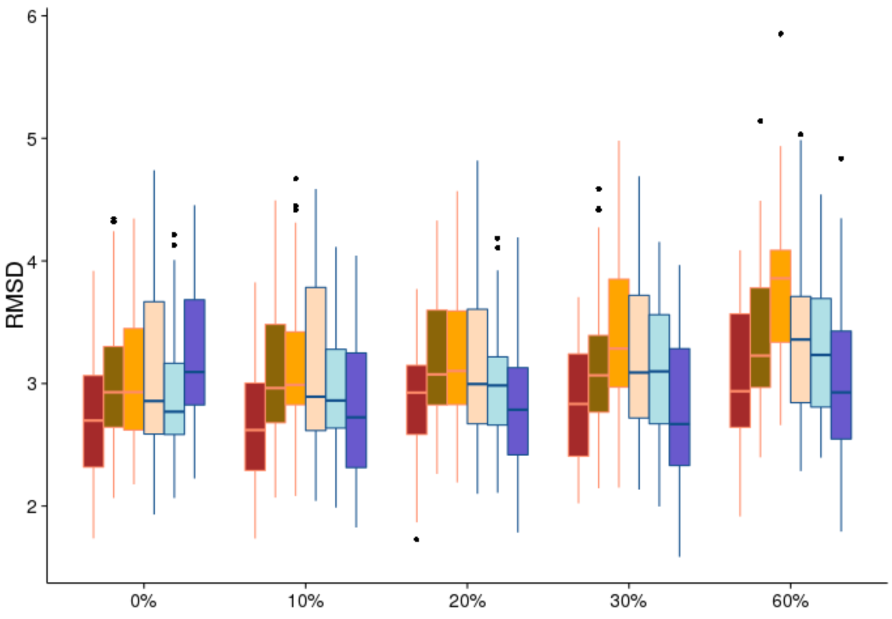

(a)

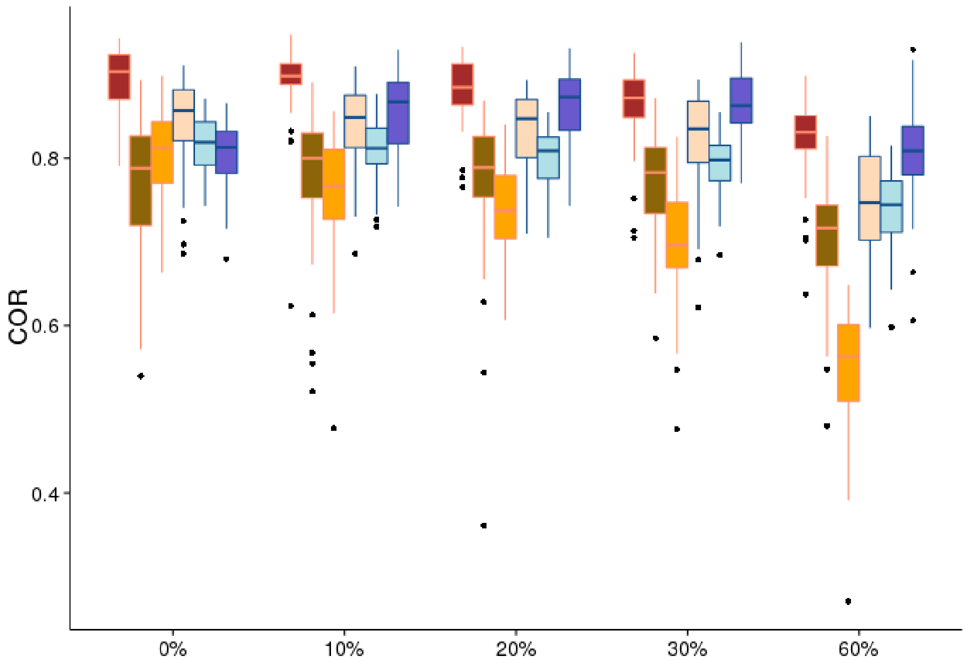

(b)

Supplementary Figure S4: Boxplots for comparing 3D estimation accuracy of six methods under ST model and parameter setting $(\beta_0, \beta_1, \gamma_1, \gamma_2) = (3, -0.434, 0.05, 0.25)$. The comparison are for data simulated from the ST model based on two criteria: (a) RMS D, and (b) Correlation. For each resolution/percent zeros, the six boxplots are for tREX, tPAM, BACH, ShRec3D, ChromSDE, and PASTIS, in that order.

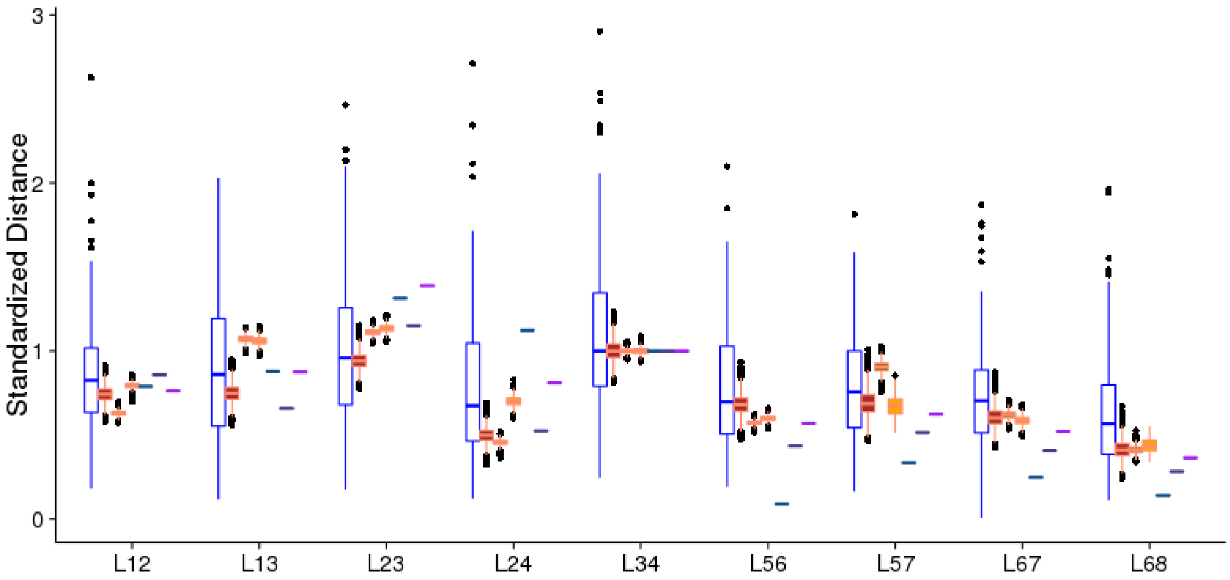

Supplementary Figure S5: Comparison of the estimated distances with the FISH measurements (gold standard) based on analyses of human lymphoblastoid cell line Hi-C data. For each pair of loci, the four boxplots are for FISH, tREX, tPAM, and BACH, in that order. More specifically, for FISH, the boxplot is based on 100 FISH measurements. For tREX, tPAM, and BACH, each boxplot is based on 10000 reconstructions of the underlying 3D structure. The line within each box represents the median; the two end points of the box mark the 25th and 75th percentiles of the measurements (for FISH) or the estimates (for tREX, tPAM, and BACH). For ShRec3D, ChromsSDE, and PASTIS, only one consensus 3D structure is reconstructed, as such their boxplot degenerates into a single line. Specifically, the three lines are for the single estimate of distance for ShRec3D, ChromSDE, and PASTIS, respectively.
